# Association Between a Co-Designed Dashboard and Use of Costly Health Services in Patients With Chronic Kidney Disease and Advanced Cancer: Propensity Score–Adjusted Difference-in-Differences Study

**DOI:** 10.2196/70430

**Published:** 2025-11-21

**Authors:** Saki Amagai, Alexandra Harris, Nisha Mohindra, Sheetal Kircher, Jeffrey A Linder, Vikram Aggarwal, John D Peipert, Katy Bedjeti, Quan Mai, Cynthia Barnard, Ava Coughlin, Mary O'Connor, Victoria Morken, David Cella, Neil Jordan

**Affiliations:** 1 Northwestern University Feinberg School of Medicine Chicago, IL United States

**Keywords:** health care cost, shared decision-making, patient-reported outcome, PRO, dashboard, PRO-based dashboard

## Abstract

**Background:**

The US health care system faces escalating costs, increasing emphasis on patient autonomy, and a regulatory shift toward patient-centered care and patient-reported outcomes (PROs). Leveraging PROs to support shared decision-making has the potential to improve outcomes and reduce health care use for patients with advanced chronic conditions.

**Objective:**

This study aims to evaluate the impact of a PRO-based clinical dashboard on the use of costly health services among patients with advanced cancer and chronic kidney disease (CKD).

**Methods:**

We conducted a quasi-experimental, propensity score–weighted, difference-in-differences analysis using routinely collected data (June 2020 to January 2022) from a large US academic health system. Dashboard users were compared with contemporaneous nonexposed patients matched on clinical criteria. The primary outcomes were unplanned all-cause hospital admissions, potentially avoidable emergency department visits, excess days in acute care within 30 days of discharge, and 7-day readmissions. Cancer-specific secondary outcomes included acute encounters during outpatient chemotherapy, oncology triage use, advance directive completion, and hospice use. CKD-specific outcomes were CKD-related acute care use and disease progression.

**Results:**

In the advanced cancer cohort (dashboard users: n=284; dashboard nonusers: n=917), dashboard use was associated with significantly fewer chemotherapy-related emergency department or hospital encounters (ratio-in–odds ratios 0.35, 95% CI 0.16-0.75) and a nonsignificant 1.7–percentage point reduction in unplanned admissions (β=–0.017, 95% CI –0.107 to 0.072). Using Firth penalized logistic regression to reduce small sample bias, dashboard use was also associated with significantly higher odds of 7-day readmissions (ratio-in–odds ratios 8.58, 95% CI 2.28-32.32). Among readmissions in the dashboard user group, most (13/14, 93%) were scheduled by clinicians. Excess days in acute care increased by 4 percentage points (β=0.040, 95% CI –0.001 to 0.089). Advance directive completion declined significantly (β=–0.009, 95% CI –0.039 to 0.020). In the CKD cohort (dashboard users: n=365; dashboard nonusers: n=2137), no significant differences were observed for any primary or CKD-specific outcome.

**Conclusions:**

In routine oncology practice, a PRO dashboard was associated with fewer acute care encounters during chemotherapy but more planned early readmissions. The dashboard had no measurable effect on patients with CKD. These disease-specific mixed results highlight the need to tailor dashboards to the clinical context and embed them within workflows that can act on real-time PRO information.

## Introduction

By 2031, health care expenditures in the United States are expected to approach 20% of the gross domestic product, surpassing other high-income nations with comparable clinical resources [1]. A significant driver of these costs is the fee-for-service payment model, which incentivizes the use of costly health care resources and services, even though they may not improve patient outcomes or quality of life. The Medicare Access and Children’s Health Insurance Program Reauthorization Act of 2015 marked a pivotal transition from a volume-oriented to value-based payment model for health care services [2-4]. Concurrently, the Centers for Medicare and Medicaid Services have prioritized development of patient-centered measures, incorporating patient-reported outcomes (PROs), such as the Patient-Reported Outcomes Measurement Information System (PROMIS), into quality evaluation and pay-for-performance programs [5].

These changes are important because patients and clinicians increasingly grapple with rising health care costs while striving to maintain high-quality care. The burden of health care expenses disproportionately affects patients with chronic, complex diseases such as advanced cancer and chronic kidney disease (CKD) [6-9]. These patients often experience distressing symptoms like fatigue, pain, anxiety, and depression [10-14]. These symptoms frequently go unnoticed during routine visits, leading to unmanaged symptoms and a greater likelihood of potentially avoidable health care resource use [15-19]. Financial strain has also been linked to increased patient debt and bankruptcy [20-22].

Given this financial strain, patients may be more willing to engage in shared decision-making (SDM) with clinicians, collaboratively weighing the benefits and risks of treatment options to align health care decisions with their preferences and values [23-25]. The growing engagement with digital health care presents an opportunity for innovative health IT solutions, such as PRO-based clinical dashboards that provide decision-making information in a format that benefits both patients and clinicians. These dashboards track clinical and health outcome trends over time, potentially reducing the risk of unplanned or low-benefit health services use by enabling early intervention in symptom management and fostering SDM discussions about bothersome side effects and treatment alternatives [24,26,27]. Prior studies have suggested that early symptom management may reduce acute care use and improve quality of life [17,28,29]. We hypothesized that PRO-based clinical dashboards could empower patients with complex diseases to visualize the relationship between interventions and outcomes, thereby reducing unnecessary spending while delivering high-value care more effectively [30].

Although PROs and SDM have been shown to be effective in symptom monitoring for patients with cancer and CKD, their influence on reducing the use of potentially unnecessary health services of limited benefit is less understood [17,28,29,31,32]. Our previous work showed that a PRO-based dashboard enhanced SDM and reduced patient anxiety [33], yet the specific role of SDM in reducing health care use remains unclear. While some studies suggest that SDM tools may reduce health care use, a Cochrane review [27,34] found mixed results, with inconsistent effects on use, outcomes, and costs, and no consistent reduction in invasive or expensive treatments [35].

In this study, we focus on the potential for PRO-based dashboards to influence the use of potentially unnecessary, expensive, and low-benefit health care services, improving SDM, enhancing symptom management, and engaging patients in care optimization. We hypothesize that patients using the dashboard will show reduced use of these services compared to a matched cohort not exposed to the dashboard [36].

## Methods

### Dashboard Design, Content, and Integration With Clinical Workflow

The development and integration of the dashboard, including its co-design process, visual elements, included assessments, and the ways physicians used it, have been comprehensively described elsewhere [33,37]. Briefly, the dashboard was co-designed with the collective effort of 20 diverse stakeholders, including patients, clinicians, care partners, investigators, and health IT professionals. The goal was to support symptom management and facilitate SDM during health care visits for patients with advanced cancer or CKD. Integrated into Northwestern Medicine’s electronic health record system, the dashboard displays PROs along with other clinical data. Clinicians were encouraged to use the dashboard, updated in real time, during clinical encounters with patients who met the study’s inclusion criteria.

Three days before a scheduled visit, patients were prompted to complete a PRO questionnaire (Multimedia Appendix 1), which assessed symptoms and supportive care needs. The questionnaire included PROMIS measures to assess anxiety, depression, pain, fatigue, and physical functioning. In addition, patients responded to five open-ended questions in the “symptoms and goals” section, which populated the dashboard. These questions focused on (1) top concerns for discussion, (2) the most troubling side effects, (3) overall goals for their cancer or CKD treatment, (4) personal goals and values, and (5) potential ways to collaborate with their care team to achieve these goals. Patient responses were automatically scored, recorded in the electronic health record, and generated alerts to their care team if any clinically significant symptoms or needs were identified. Clinicians then used this information, along with the patient’s clinical data from the dashboard, to facilitate SDM and improve communication between patients and care teams.

### Study Design and Location

We conducted a propensity score–weighted, difference-in-differences (DiD) analysis [38] to determine the association between dashboard use and high-cost health services use. The study protocol has previously been published [37]. This manuscript adheres to the STROBE (Strengthening the Reporting of Observational Studies in Epidemiology) guidelines for cohort studies.

### Ethical Considerations

All study activities were reviewed and approved by the Northwestern University Institutional Review Board (protocols STU00210091, STU00211654, and STU00212634). Health services use data were extracted from the Northwestern Medicine Enterprise Data Warehouse by trained Northwestern Medicine data analysts and entered into the study’s REDCap database by the study coordinator (AC), as approved by the ethics committee. All study procedures were considered low risk by the Northwestern University Institutional Review Board, and the ethics review concluded that the benefits outweighed any minimal risks. Participants provided informed consent to complete a follow-up survey at 3 and 6 months. Participants did not receive any incentives for enrollment or survey completion.

All data were aggregated by an Enterprise Data Warehouse (EDW) analyst prior to analysis, and no protected health information (PHI) was visible to the research team. Limited variables—such as medical record number (MRN), race/ethnicity, and age—were retained solely to enable patient-level linkage and inclusion as covariates in multivariable analyses. All data were stored on secure, access-controlled institutional servers, and no identifiers were shared outside the research environment. All study procedures adhered to institutional privacy and confidentiality standards and complied with the Health Insurance Portability and Accountability Act (HIPAA) regulations.

### Participants and Eligibility Criteria

The intervention group consisted of Northwestern Medicine patients in Chicago, Illinois, diagnosed with advanced cancer or CKD between June 2020 and January 2022 who had previously received care from at least one clinician participating in the study (Multimedia Appendix 2). These clinicians at Northwestern Memorial Health Care included 2 oncologists, a nephrologist, a nephrology physician assistant, and 2 primary care physicians. Patients also consented to follow-up surveys at 3- and 6-month intervals. Patients with advanced cancer were defined as having either stage IV gastrointestinal cancer receiving intravenous chemotherapy for at least 3 months or stage III or IV lung cancer undergoing first- or second-line chemotherapy for at least 3 months. Patients with CKD required a confirmed diagnosis of at least stage III CKD or an estimated glomerular filtration rate (eGFR) below 60. For the intervention group, the baseline date was defined individually as the date on which each participant completed the initial dashboard questionnaire from June 8, 2020, to November 1, 2022. For the comparison group, we identified patients with advanced cancer and CKD not exposed to the dashboard. The inclusion criteria were patients who received care from Northwestern Medicine clinicians who participated in the dashboard study but chose not to enroll in it or patients who received care during the same time period from Northwestern Medicine clinicians who were not involved in the dashboard study.

For patients with advanced cancer in the comparison group, the baseline date was determined as the visit closest to the intervention patient’s baseline date (within 30 days) during the established time frame for the dashboard baseline (June 8, 2020, to November 1, 2022). For CKD comparison patients, the baseline was similarly defined as the first instance where their eGFR dropped below 60, adjusted to align with the intervention patients’ baseline date within the same time frame. To account for variability in eGFR, the mean baseline eGFR for each patient was calculated using eGFR values within 30 days of the baseline date for both groups. Patients with a mean eGFR above 60, indicating CKD stage II or lower, were excluded to ensure comparability in disease severity. Because this was a pragmatic, real-world evaluation, no power calculation was performed a priori. Instead, we included every patient who met the above eligibility criteria during the June 2020 to January 2022 accrual window.

### Outcome Variables

Data on the use of potentially avoidable, high-cost, or low-value health services and select metrics of appropriate care were extracted from the clinical records for the period spanning 6 months before and 6 months after each patient’s baseline date. Specific indicators included unplanned all-cause hospital admissions, potentially avoidable all-cause emergency department use, all-cause excess days in acute care (EDAC) within 30 days following hospital discharge, and 7-day readmissions. Among patients with advanced cancer, the following disease-specific indicators were also assessed: hospital admissions and emergency department visits for patients receiving outpatient chemotherapy, use of a triage clinic, completion of an advance directive, and any hospice use. Among patients with CKD, we also assessed the following additional indicators: CKD-related emergency department or hospital inpatient use and progression from CKD stage III to stage IV, stage IV to stage V, or stage III to stage V.

### Statistical Analyses

#### DiD Framework

We used a DiD approach to assess changes in the use of high-cost health services and select metrics of appropriate care between patients in the dashboard groups and those in the comparison groups. All analyses were run separately for each disease cohort (advanced cancer and CKD) to account for their distinct clinical trajectories and use patterns. Health services use in the 6 months before the dashboard intervention were compared with use during the 6 months after the intervention, with the following equation:

*y* = *β*_0_ + *β*_1_ ∙ *Time* + *β*_2_ ∙ *Treated* + *β*_3_ ∙ (*Time* × *Treated*) + *β*_4_ ∙ *Covariates* + *ε*

An interaction term for intervention group (“treated”) and time period (“time”) was included to test whether changes from the preintervention to postintervention periods differed between the dashboard and comparison groups. A statistically significant effect for the time period × intervention group interaction term (*β*_3_) would suggest that the dashboard intervention is associated with differential health services use. The DiD models were estimated with Huber-White cluster-robust SEs that correct for heteroskedasticity, account for paired observations within patients, and cluster at the health care provider level to absorb unmeasured health care provider–level confounding [39].

#### Propensity Scores and Inverse-Propensity Weighting

A valid DiD estimate rests on the *parallel trends* assumption—that in the absence of the intervention, average outcomes in the dashboard and comparison groups would have evolved similarly over time. Because our dataset included a single preintervention measurement for each outcome, we could not empirically test for parallel preperiod slopes. Instead, we increased the plausibility of a *conditional* parallel trends assumption by ensuring that the two groups were closely matched on observed baseline characteristics. Specifically, we estimated propensity scores [40,41] using a logistic regression model that included race, ethnicity, age, sex, insurance category, Charlson Comorbidity Index, baseline encounter date, median household income, and baseline health services use (number of observation admissions, number of outpatient encounters, and number of immediate urgent care encounters). Median household income, used as a proxy for socioeconomic status, was obtained from 5-digit residential zip codes in the Social Determinants of Health Database from the Agency for Healthcare Research and Quality [42]. Cancer type was added as a covariate to the advanced cancer propensity score model, and CKD stage was included in the CKD propensity score model. Dashboard patients received a weight equal to 1/(propensity score), whereas comparison patients were weighted as 1/(1 – propensity score). Weights greater than 10 were truncated to reduce the influence of outliers. The weighted analysis, therefore, estimated the average treatment effect on the treated (ATT).

#### Primary Model: Weighted Linear Probability DiD

We chose a linear probability model for ease of interpretation. The resulting β coefficients reflect absolute percentage point changes (eg, β=–0.05 implies a 5–percentage point reduction in risk for the dashboard group relative to the comparison group). Cancer type (advanced cancer cohort) or CKD stage (CKD cohort) was included as a fixed effect. Hospice use and CKD progression were observed only after the intervention; these outcomes were analyzed with single period–weighted linear probability models that omitted the period variable. Unadjusted models are reported in Multimedia Appendices 3 and 4.

### Robustness for Low-Prevalence Outcomes

Some outcomes (eg, 7-day admission) have prevalences less than 20%. We, therefore, reestimated each model with the same inverse propensity weights using a logistic DiD model; the exponentiated interaction coefficient is reported as the ATT ratio-in–odds ratios (ROR). Linear ATT and logit ATT RORs are presented in the Results section, and the two specifications produce directionally and statistically consistent results. For very low sample prevalence (<10%), we additionally applied the Firth penalized likelihood method to mitigate small sample bias (Multimedia Appendix 5) [43]. For readers interested in population-level effects, a set of inverse propensity–weighted linear probability models estimating the average treatment effect in the entire study population is also provided in Multimedia Appendices 6 and 7.

### Software and Packages

All statistical analyses were conducted in R (version 4.2.1; R Foundation for Statistical Computing) using *sandwich* 3.0.2 (robust variance) and *WeightIt* 1.4.0 (inverse propensity score weighting).

### Hypothesis

We hypothesized that patients in the dashboard group would have fewer unplanned all-cause hospital admissions, fewer EDACs, and lower 7-day readmission rates than patients in the comparison cohort. For the cancer dashboard group, we anticipated higher rates of oncology triage clinic visits, advance directive completion, and hospice use, with a reduced likelihood of chemotherapy within the last 14 days of life. For the CKD dashboard group, we expected a decrease in emergency-start dialysis, CKD-related emergency department or inpatient use, and slower progression of CKD stages.

## Results

### Study Population

**Figure 1:**
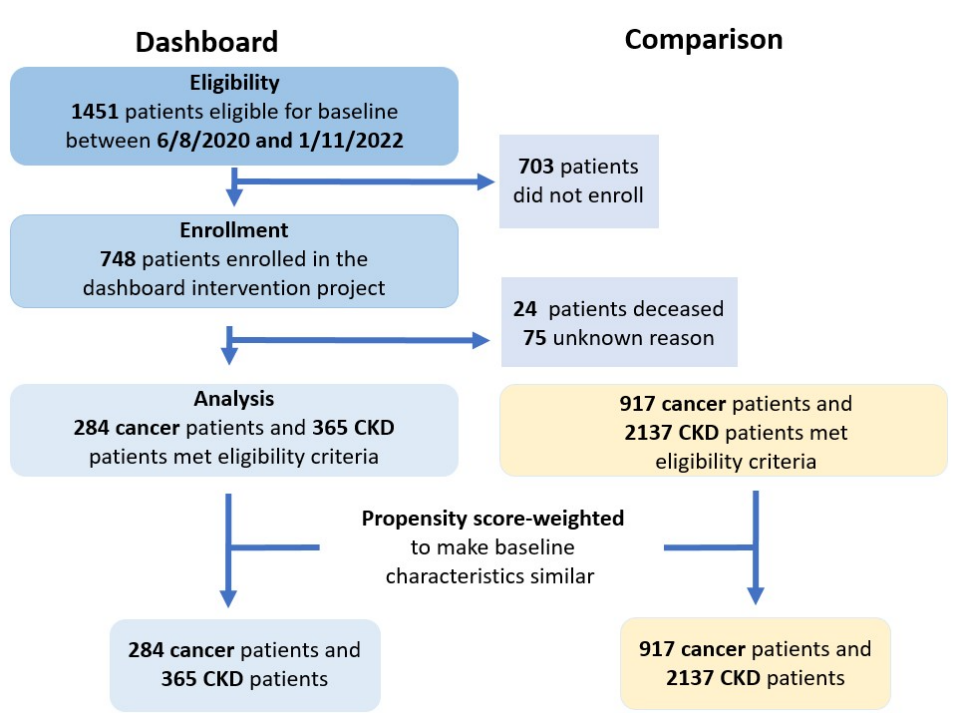
Study flowchart of dashboard and comparison group selection. CKD: chronic kidney disease.

Of the 748 patients enrolled in the dashboard study, 284 patients with advanced cancer and 365 patients with CKD completed the baseline questionnaires and composed the dashboard cohorts (Figure 1). The comparison cohorts consisted of 917 patients with advanced cancer and 2137 patients with CKD who met the eligibility criteria but were not exposed to the dashboard. Before weighting, several baseline characteristics differed statistically between the dashboard and comparison groups (Tables 1 and 2). For example, the patients with advanced cancer in the dashboard group had more comorbidities (mean Charlson Comorbidity Index 8.62, SD 3.22, vs 6.93, SD 3.34) and were more often treated for lung cancer (191/284, 67.3% vs 176/917, 19.2%). Among patients with CKD, there were significant differences between the dashboard and comparison groups in CKD stage (*P*=0.005) and in the number of inpatient (*P*<0.001) and outpatient encounters (*P*<0.001).

**Table 1:** Cancer cohort: comparisons of demographic characteristics, previous health care use, and comorbidities between dashboard and comparison groups.

		Unweighted								Weighted^a^						
		Dashboard (n=284)	Comparison (n=917)	SMD^b,c^		*P* value		Dashboard			Comparison		SMD^c^		*P* value	
**Race, n (%)**					0.130		.05							0.067		.79
	White	189 (66.5)	628 (68.5)					68.8			68.2					
	Black	46 (16.2)	109 (11.9)					13.5			12.6					
	Declined to answer	17 (6.0)	61 (6.7)					6.9			6.5					
	Other	32 (11.3)	119 (13.0)					10.7			12.7					
**Ethnicity, n (%)**					0.186		.002							0.090		.50
	Non-Hispanic	253 (89.1)	774 (84.4)					82.2			85.3					
	Declined to answer	23 (8.1)	82 (8.9)					10.2			9.0					
	Hispanic	8 (2.8)	61 (6.7)					7.6			5.7					
Charlson Comorbidity Index, mean (SD)		8.62 (3.22)	6.93 (3.34)	0.52		<.001		7.59 (3.23)			7.40 (3.54)		0.057		.38	
Age (years), mean (SD)		63.12 (12.84)	62.04 (12.66)	0.085		.08		63.37 (12.49)			62.48 (12.38)		0.072		.25	
**Insurance, n (%)**					0.194		.002							0.111		.34
	Commercial	135 (47.5)	480 (52.3)					51.5			51.5					
	Medicaid	15 (5.3)	74 (8.1)					5.1			7.1					
	Medicare	134 (47.2)	360 (39.3)					43.4			41.2					
	Uninsured	0 (0.0)	3 (0.3)					0.0			0.2					
Male, n (%)		130 (45.8)	476 (51.9)	0.123		.01		48.1			50.4		0.046		.49	
Lung cancer, n (%)		191 (67.3)	176 (19.2)	1.109		<.001		32.9			31.4		0.032		.58	
Emergency encounters (n), mean (SD)^d^		1.15 (1.72)	0.83 (1.48)	0.203		<.001		1.15 (1.68)			0.92 (1.54)		0.146		.03	
Observation hospital admissions (n), mean (SD)^d^		0.20 (0.55)	0.18 (0.54)	0.038		.42		0.27 (0.72)			0.19 (0.55)		0.134		.10	
Inpatient encounters (n), mean (SD)^d^		0.78 (1.26)	0.59 (1.04)	0.167		<.001		0.71 (1.22)			0.62 (1.08)		0.074		.27	
Immediate urgent care encounters (n), mean (SD)^d^		0.54 (1.12)	0.37 (0.98)	0.153		.001		0.53 (1.15)			0.43 (1.07)		0.094		.17	
Outpatient encounters (n), mean (SD)^d^		25.44 (18.69)	14.98 (15.41)	0.611		<.001		20.42 (14.64)			17.72 (17.32)		0.168		.002	
Median household income (US $), mean (SD)		85,720 (32,862)	86,292 (31,095)	0.179		.71		85,285 (32,921)			85,873 (30,875)		0.018		.79	

^a^The sum of weights for the dashboard group was 1135. The sum of weights for the comparison group was 1212.45. Previous health care use indicates health care services used during the 12 months prior to the baseline assessment.

^b^SMD: standardized mean difference.

^c^SMD quantifies the difference in a covariate’s mean values between groups, scaled by the pooled SD. While SMD values closer to 0 suggest better balance, values over 0.1 may indicate potential differences. In this study, most covariates demonstrated good balance after weighting, although a small number retained statistical significance at *P*<.05. To mitigate potential residual differences, the difference-in-differences outcome model included unit and time fixed effects and controlled for all baseline covariates regardless of their *P* values.

^d^Encounters that occurred 12 months prior to baseline were included.

**Table 2:** Chronic kidney disease (CKD) cohort: comparisons of demographic characteristics, previous health care use, and comorbidities between the dashboard and comparison groups.

		Unweighted								Weighted^a^						
		Dashboard (n=365)	Comparison (n=2137)	SMD^b,c^		*P* value		Dashboard			Comparison		SMD^c^		*P* value	
**Race, n (%)**					0.092		.19							0.050		.75
	White	183 (50.1)	1036 (48.5)					46.6			48.6					
	Black	120 (32.9)	674 (31.5)					33.8			31.8					
	Declined to answer	15 (4.1)	125 (5.8)					6.0			5.6					
	Other	47 (12.9)	302 (14.1)					13.6			13.9					
**Ethnicity, n (%)**					0.101		.05							0.024		.87
	Non-Hispanic	312 (85.5)	1753 (82.0)					81.6			82.5					
	Declined to answer	19 (5.2)	121 (5.7)					6.0			5.6					
	Hispanic	34 (9.3)	263 (12.3)					12.4			11.9					
Charlson Comorbidity Index, mean (SD)		8.38 (3.79)	7.73 (3.69)	0.173		<.001		7.85 (3.65)			7.82 (3.81)		0.008		.85	
Age (years), mean (SD)		64.31 (14.10)	63.46 (15.97)	0.056		.18		64.08 (14.42)			63.54 (15.90)		0.036		.40	
**Insurance, n (%)**					0.058		.72							0.055		.55
	Commercial	131 (35.9)	744 (34.8)					34.2			35.0					
	Medicaid	33 (9.0)	192 (9.0)					9.6			9.0					
	Medicare	201 (55.1)	1198 (56.1)					56.2			56.0					
	Uninsured	0 (0.0)	3 (0.1)					0.1			0.0					
Male, n (%)		209 (57.3)	1066 (49.9)	0.148		<.001		51.7			50.9		0.017		.70	
Emergency encounters (n), mean (SD)^d^		1.58 (2.23)	1.37 (2.60)	0.086		.04		1.62 (2.48)			1.40 (2.63)		0.087		.15	
Observational encounters (n), mean (SD)^d^		0.38 (0.71)	0.29 (0.70)	0.126		.002		0.32 (0.66)			0.31 (0.71)		0.025		.55	
Inpatient encounters (n), mean (SD)^d^		1.05 (1.51)	0.72 (1.35)	0.229		<.001		0.80 (1.25)			0.77 (1.41)		0.021		.62	
Immediate urgent care encounters (n), mean (SD)^d^		0.73 (1.63)	0.64 (2.03)	0.046		.28		0.69 (1.60)			0.66 (2.07)		0.019		.51	
Outpatient hospital admissions (n), mean (SD)^d^		20.69 (15.39)	15.78 (14.31)	0.331		<.001		17.10 (13.08)			16.73 (16.44)		0.025		.51	
Median household income (US $), mean (SD)		77,925 (30,283)	77,432 (31,131)	0.016		.69		77,401.71 (30,239)			77,505.09 (31,146)		0.034		.94	
**CKD stage, n (%)**					0.129		.005							0.037		.68
	Stage III	54.8	61.1					58.3			60.1					
	Stage IV	30.1	26.2					27.7			26.7					
	Stage V	15.1	12.7					14.0			13.2					

^a^The sum of weights for the dashboard group was 2504.1. The sum of weights for the comparison group was 2504.85. Previous health care use indicates health care services used during the 12 months prior to the baseline assessment.

^b^SMD: standardized mean difference.

^c^SMD quantifies the difference in a covariate’s mean values between groups, scaled by the pooled SD. While SMD values closer to 0 suggest better balance, values over 0.1 may indicate potential differences. In this study, *P* values were also assessed, demonstrating minimal statistical differences between groups, even for covariates with SMD values close to 0.1. To mitigate potential residual differences, the difference-in-differences outcome model included unit and time fixed effects and controlled for all baseline covariates regardless of their *P* values.

^d^Encounters that occurred 12 months prior to baseline were included.

After applying inverse propensity weighting, the standardized mean difference was less than 0.10 for every variable except outpatient encounter volume in the cancer cohort. Accordingly, the covariates were included as fixed effects in the weighted DiD models to further control for potential confounding and improve comparability (Tables 1 and 2) [38,44].

### Association Between Dashboard Use and High-Cost Services Use

#### Advanced Cancer Cohort

In weighted DiD models, dashboard exposure was not associated with changes in unplanned all-cause admission rates (ATT: β=–0.017, 95% CI –0.107 to 0.072; ROR 0.89, 95% CI 0.46-1.72; Table 3). In contrast, dashboard exposure was associated with a 4–percentage point increase in EDACs relative to the comparison group (ATT: β=0.040, 95% CI –0.001 to 0.089), although the corresponding odds ratio was not significant (ROR 5.84, 95% CI 0.89-38.42). Dashboard users also experienced a 3.7–percentage point rise in the 7-day readmission rate (ATT: β=0.037, 95% CI 0.008-0.066), with a significant increase in odds (Firth-adjusted ROR 8.58, 95% CI 2.28-32.32). Chart review confirmed that 93% of these early readmissions were scheduled by clinicians, suggesting intentional proactive care rather than unplanned deterioration.

**Table 3:** Cancer: propensity score–weighted difference-in-differences results^a^.

Health services type	Dashboard group (n=284), n (%)			Comparison group (n=917), n (%)			Linear ATT^b^, b (95% CI)		Logit ATT, ROR^c^ (95% CI)
	Before	After	Before		After				
Unplanned all-cause hospital admissions	57 (20.1)	63 (22.1)	157 (17.1)		244 (26.7)	–0.017 (–0.107 to 0.072)		0.892 (0.463 to 1.720)	
EDAC^d^ within 30 days of hospital discharge	116 (40.8)	129 (45.4)	143 (15.6)		219 (23.9)	0.040 (–0.001 to 0.089)		5.838 (0.887 to 38.424)	
7-day hospital readmissions	4 (1.4)	14 (4.9)	4 (0.4)		5 (0.5)	0.037 (0.008 to 0.066)		9.544 (1.339 to 60.192)	
Hospital admissions and ED^e^ visits for patients receiving outpatient chemotherapy	30 (10.6)	54 (19.0)	43 (4.7)		161 (17.6)	–0.014 (–0.102 to 0.074)		0.351 (0.163 to 0.753)	
Oncology triage clinic use	40 (14.1)	56 (19.7)	32 (3.4)		80 (8.7)	0.047 (–0.031 to 0.125)		0.674 (0.322 to 1.412)	
Completion of an advanced directive	5 (1.7)	5 (1.7)	11 (1.2)		33 (3.6)	–0.009 (–0.039 to 0.020)		0.245 (0.048 to 1.247)	
Hospice use^f^	—^g^	11/27 (40.7)	—		32/65 (49.2)	0.203 (–0.049 to 0.454)		2.837 (0.755 to 10.662)	

^a^All coefficients are the ATT (b) obtained with inverse propensity-weighted difference-in-differences modes. Linear ATT is the treatment-effect coefficient from the weighted linear probability DiD; logit ATT ROR is the corresponding ratio-in–odds ratios from a weighted logistic regression fit to the identical analytic sample. Both models adjust for all baseline covariates included in the propensity score specification to minimize residual confounding: race, ethnicity, age, sex, insurance category, Charlson Comorbidity Index, cancer type, baseline use encounter date, median household income (zip code level), and baseline use counts (emergency, observation, inpatient, immediate/urgent care, and outpatient encounters). Time and time × treated interaction terms were excluded from the regression analyses.

^b^ATT: average treatment effect on the treated.

^c^ROR: ratio-in–odds ratios.

^d^EDAC: excess days in acute care.

^e^ED: emergency department.

^f^For hospice use, the denominator is restricted to patients who died during the study period who received care from a participating study physician.

^g^Not applicable.

Notably, the pattern reversed among patients receiving outpatient chemotherapy. The dashboard intervention was associated with a significant 65% reduction in the odds of acute care use (hospital admission or emergency department visit) in this subgroup (ROR 0.35, 95% CI 0.16-0.75), although the linear DiD estimate did not reach statistical significance. Conversely, completion of advance directives declined among dashboard users (Firth-adjusted ROR 0.25, 95% CI 0.10-0.67). No statistically significant associations were observed for oncology triage visits or hospice use.

#### CKD Cohort

In the CKD cohort, dashboard exposure was not associated with any of the prespecified outcomes, including unplanned admissions, EDACs, 7-day readmissions, CKD-related acute use, or CKD progression, under either the linear ATT β or Firth-adjusted logit ATT ROR specification (Table 4).

**Table 4:** Chronic kidney disease (CKD): propensity score–weighted difference-in-differences results^a^.

Health services type	Dashboard group (n=365), n (%)		Comparison group (n=2137), n (%)		Linear ATT^b^, b (95% CI)	Logit ATT, ROR^c^ (95% CI)
	Before	After	Before	After		
Unplanned all-cause hospital admissions	112 (30.7)	94 (25.8)	412 (19.3)	385 (18.0)	–0.010 (–0.073 to 0.053)	0.879 (0.565 to 1.368)
EDAC^d^ within 30 days of hospital discharge	20 (5.4)	22 (6.0)	32 (1.5)	23 (1.1)	0.001 (–0.035 to 0.036)	1.300 (0.491 to 3.442)
7-day hospital readmissions	7 (1.9)	9 (2.4)	12 (0.6)	10 (0.5)	0.006 (–0.016 to 0.028)	1.591 (0.364 to 6.950)
CKD-related ED^e^ or inpatient use	128 (35.1)	112 (30.7)	483 (22.6)	523 (24.5)	–0.050 (–0.118 to 0.018)	0.752 (0.502 to 1.126)
Progression from CKD stage III to IV, stage IV to V, and stage III to stage V	—^f^	31 (8.4)	—	334 (15.6)	–0.002 (–0.034 to 0.029)	1.073 (0.695 to 1.655)

^a^All coefficients are the ATT obtained with inverse propensity weighted difference-in-differences modes. Linear ATT (b) is the treatment effect coefficient from the weighted linear probability difference in differences; logit ATT ROR is the corresponding ratio-in–odds ratios from a weighted logistic regression fit to the identical analytic sample. Both models adjust for all baseline covariates included in the propensity score specification to minimize residual confounding: race, ethnicity, age, sex, insurance category, Charlson Comorbidity Index, CKD stage, baseline encounter date, median household income (zip code level), and baseline use counts (emergency, observation, inpatient, immediate/urgent care, and outpatient encounters). Time and time × treated interaction terms were excluded from the regression analyses.

^b^ATT: average treatment effect on the treated.

^c^ROR: ratio-in–odds ratios.

^d^EDAC: excess days in acute care.

^e^ED: emergency department.

^f^Not applicable.

## Discussion

### Principal Findings

Using a propensity score–weighted DiD design, we found that the co-designed PRO dashboard had disease-specific and mixed effects on use of high-cost services. For patients with advanced cancer, dashboard use was associated with fewer acute encounters during outpatient chemotherapy (a 65% reduction in odds), yet dashboard use also coincided with increased planned 7-day readmissions and a modest rise in excess days in acute care. No other use outcomes changed, and no effects were detected in the CKD cohort.

### Comparison With Prior Work

Previous randomized symptom monitoring trials in oncology have reported 30% to 50% reductions in emergency department visits and hospitalizations when PRO alerts triggered nurse triage or oncologist feedback [45,46]. Our observed 65% reduction in odds of acute care encounters related to outpatient chemotherapy aligns with these findings, suggesting that PRO-informed dashboards can be effective in real-world clinical settings, not just trials. An increase in 7-day readmission rates mirrors patterns seen in heart failure programs, where early, proactive readmissions are reframed as planned care. This suggests that the dashboard may support clinician-directed early symptom management.

We also observed a significant decrease in advance directive completion among dashboard users in the advanced cancer cohort. This may reflect a shift of clinical attention toward pressing symptoms, similar to a US Department of Veterans Affairs trial where increased planning discussions did not improve alignment with patient preferences [15]. This highlights how even well-designed dashboards can induce unintended consequences, underscoring the need for thoughtful integration into care processes.

For CKD, our null findings align with a systematic review indicating that stand-alone SDM tools rarely impact high-cost use metrics [47]. Without concurrent disease management supports or stronger engagement by clinicians and patients, dashboards may be insufficient to change care trajectories in slowly progressive conditions. Current evidence, therefore, remains mixed on whether SDM interventions alone can consistently curb costly service use.

### Implications for SDM Tools

Patient engagement in SDM is shaped by multiple forces. While financial strain can sharpen patients’ desire to weigh costs against benefits, the general shift toward patient-centered care, greater transparency in outcomes, and heightened emphasis on quality of life also motivate participatory decisions [47]. Our dashboard, which pairs PRO trends with open-ended prompts on the goals of care, may have resonated most strongly with oncology patients because acute toxicity and quality of life trade-offs are immediate and visible during chemotherapy. CKD trajectories are typically slower; without real-time laboratory or symptom triggers, the dashboard information may have seemed less actionable to nephrology teams, resulting in lower engagement and impact in that setting.

### Cancer-Type Heterogeneity

The advanced cancer cohort comprised patients with lung and gastrointestinal malignancies. Although both subgroups face heavy symptom burdens, needs can diverge: patients with lung cancer report dyspnea and cough as dominant concerns, whereas patients with gastrointestinal cancer often prioritize nausea, appetite, and bowel symptoms [48-50]. The dashboard displayed all PROMIS domains identically, which may have diluted its relevance for patients whose dominant symptoms were not being addressed in the clinic. Future iterations could include cancer-specific symptom widgets or algorithmic highlighting of domain scores most relevant to each cancer type to increase salience for both patients and health care providers.

### Strengths and Limitations

The study’s key strengths are its evaluation in routine clinical practice across two distinct specialties (oncology and nephrology), use of rigorous inverse propensity weighting that achieved robust covariate balance, and parallel reporting of additive (percentage point) and multiplicative (ROR) effects, which allows findings to be interpreted consistently across outcomes with different prevalence rates.

There are several limitations. First, all use data were drawn from a single US academic health network; encounters that occurred elsewhere were unseen, and results may not generalize to community or non-US settings. Second, patients were not randomly assigned to the dashboard intervention, which limits our ability to make causal claims about the effect of dashboard use. Although our weighting approach improved the balance of patient characteristics between the intervention and comparison groups, residual confounding from unmeasured factors (eg, digital literacy) could remain. Third, dashboard patients exhibited higher baseline use, suggesting higher initial health care needs; the analytical approach mitigated but may not have fully eliminated this imbalance, potentially biasing postintervention contrasts.

### Conceptual Implications for Dashboard Design

Our findings reinforce a fundamental principle of user-centered, participatory design: a dashboard’s value depends on how well it integrates with the downstream clinical workflow. In our advanced cancer cohort, the dashboard was associated with fewer chemotherapy-related acute care encounters, suggesting that when clinicians have a clear, rapid way to respond to symptom data, visualizing those data in a dashboard may avert costly health services use. In contrast, the absence of any effect among patients with CKD may imply that, without an equally responsive care pathway, a stand-alone dashboard cannot produce meaningful change.

This observation aligns with evidence from a recent participatory design intervention in surgical ward rounds, where stakeholders confirmed that successful implementation hinged on both tool usability and system readiness, including routines, coordination, and technology infrastructure [51]. Just as that study revealed the importance of aligning design with contextual factors and cultural norms, our dashboard’s impact depended on having disease-specific support pathways in place.

Future versions should, therefore, be co-designed not only around what information is displayed but also around disease-specific response workflows (eg, automated alerts routed to the appropriate clinician, or prompts that trigger standing orders) [47].

### Conclusions

While a co-designed dashboard may help reduce high-cost health services use and improve select care metrics for patients with advanced cancer, the dashboard appears to be less effective for patients with CKD. As the first study on an SDM intervention and its impact on health services use for these groups, the results were mixed. More research is needed to fully understand the impact of co-designed dashboards on improving emotional, clinical, and use outcomes.

## Data Availability

The data supporting these study findings were derived from electronic health records within the participating health care system and are not publicly available due to privacy and confidentiality restrictions.

## Appendix

Multimedia Appendix 1 Previsit questionnaires.

Multimedia Appendix 2 Group definitions: dashboard vs comparison group.

Multimedia Appendix 3 Cancer: propensity score–weighted difference-in-differences results.

Multimedia Appendix 4 Chronic kidney disease: unweighted difference-in-differences results.

Multimedia Appendix 5 Propensity score–weighted difference-in-differences results (average treatment effect on the treated) with Firth penalized likelihood method.

Multimedia Appendix 6 Cancer: propensity score–weighted differences-in-difference results (average treatment effect in the entire study population).

Multimedia Appendix 7 Chronic kidney disease: propensity score–weighted difference-in-differences results (average treatment effect in the entire study population).

Multimedia Appendix 8 STROBE checklist.

## Abbreviations

ATT: average treatment effect on the treated
CKD: chronic kidney disease
DiD: difference in differences
EDAC: excess days in acute care
eGFR: estimated glomerular filtration rate
PRO: patient-reported outcome
PROMIS: Patient-Reported Outcomes Measurement Information System
ROR: ratio-in–odds ratios
SDM: shared decision-making
STROBE: Strengthening the Reporting of Observational Studies in Epidemiology
