# Supplementary material for: Association Between a Co-Designed Dashboard and Use of Costly Health Services in Patients With Chronic Kidney Disease and Advanced Cancer: Propensity Score–Adjusted Difference-in-Differences Study
Source: J Med Internet Res. 2025 Nov 21;27:e70430. doi: 10.2196/70430 (PMC12680935; doi:10.2196/70430)
Supplement: Multimedia Appendix 1 [file jmir_v27i1e70430_app1.docx]

**CANCER**

1. **Appointment Agenda—What do you want to make sure to discuss in this clinic visit? (Top 1-2 concerns)**
2. **Appointment Agenda—What are your most concerning side effects now (if any)?**
3. **Health Goals—What are you hoping we can achieve with your treatment?**
4. **Health Goals—What are some major personal goals or values you want your team to be aware of?**
5. **Health Goals—How can we work together to help you achieve your goals?**
6. **Care partner designation—If you have a care partner (a person supporting you in your care planning), please list their first and last name to allow them to appear as a member of your “care team” on the dashboard.**
7. **Care partner designation—*Please indicate this person’s relationship to you. E.g.: “This person is my…”(spouse, child, parent, friend, other)***
8. **Needs Assessment—I would like a social worker to contact me for help with (check all that apply):**

0= No needs at this time
1= Transportation resources
2= Support to help me cope with my illness and/or manage stress
3= Advance directives: medical actions to be taken if my health prevents me from making decisions (living will, power of attorney)
4= Financial concerns about my health care

1. **Needs Assessment—I would like a dietician to contact me to help with (check all that apply):**

0= No nutrition needs at this time
1= Significant gain or loss of weight without trying
2= Loss of appetite
3= Nausea / vomiting
4= Diarrhea
5= Constipation
6= Difficulty chewing or swallowing
7= Issues with my ostomy / feeding tube

1. **FACT-G7 (Version 4), GF7—I am content with the quality of my life right now...........**

0 = Not at all 1 = A little bit 2 = Somewhat 3 = Quite a bit 4 = Very much

1. **PROMIS Global01—In general, would you say your health is...........**

5 = Excellent 4 = Very good 3 = Good 2 = Fair 1 = Poor

1. **PROMIS Item Bank v1.0 - Dyspnea Severity (DYSSV014)—Over the past 7 days, how short of breath did you get with this activity….. standing for at least 5 minutes?**

0 = No shortness 1 = Mildly short 2 = Moderately short 3 = Severely short X I did not do this

of breath of breath of breath of breath in the past 7 days

1. **PRO-CTCAE22b- Severity—In the last 7 days, what was the SEVERITY of your ARM OR LEG SWELLING at its WORST?**

0 = None 1 = Mild 2 = Moderate 3 = Severe 4 = Very Severe

1. **PROMIS Scale v1.0 - Gastrointestinal Nausea and Vomiting (GISX49)—How often did you have nausea—that is, a feeling like you could vomit?**

1 = Never 2 = Rarely 3 = Sometimes 4 = Often 5 = Always

1. **PROMIS Scale v1.0 - Gastrointestinal Nausea and Vomiting (GISX55)—How often did you have a poor appetite?**

1 = Never 2 = Rarely 3 = Sometimes 4 = Often 5 = Always

1. **FACT/GOG-NTX-4 (V4) #1—I have numbness or tingling in my hands………………...**

0 = Not at all 1 = A little bit 2 = Somewhat 3 = Quite a bit 4 = Very much

1. **FACT/GOG-NTX-4 (V4) #2—I have numbness or tingling in my feet……………..**

0 = Not at all 1 = A little bit 2 = Somewhat 3 = Quite a bit 4 = Very much

1. **FACT/GOG-NTX-4 (V4) #3—I feel discomfort in my hands…………….**

0 = Not at all 1 = A little bit 2 = Somewhat 3 = Quite a bit 4 = Very much

1. **FACT/GOG-NTX-4 (V4) #4—I feel discomfort in my feet………………**

0 = Not at all 1 = A little bit 2 = Somewhat 3 = Quite a bit 4 = Very much

1. **PRO-CTCAE 15a-Severity—In the last 7 days, what was the SEVERITY of your CONSTIPATION at its WORST?**

0 = None 1 = Mild 2 = Moderate 3 = Severe 4 = Very Severe

1. **PROMIS Scale v1.0 Gastrointestinal Diarrhea 6a (GISX38)—In the past 7 days… How many days did you have loose or watery stools?**

1 = No days 2 = 1 day 3 = 2 days 4 = 3-5 days 5 = 6-7 days

1. **FACT-GP5—I am bothered by side effects of treatment.**

0 = Not at all 1 = A little bit 2 = Somewhat 3 = Quite a bit 4 = Very much

- **PROMIS Item Bank v1.0 Fatigue (CAT administration)**
- **PROMIS Item Bank v1.0 Anxiety (CAT administration)**
- **PROMIS Item Bank v1.0 Pain (CAT administration)**
- **PROMIS Item Bank v1.0 - Emotional Distress - Depression (CAT administration)**
- **PROMIS Item Bank v2.0 - Physical Function (CAT Administration)**

**KIDNEY DISEASE**

1. **Appointment Agenda—What do you want to make sure to discuss in this clinic visit? (Top 1-2 concerns)**
2. **Health Goals—What are your overall goals regarding kidney disease treatment?**
3. **Health Goals—What are some major personal goals or values you want your team to be aware of?**
4. **Health Goals—How can we work together to help you achieve your goals?**
5. **Care partner designation—If you have a care partner (a person supporting you in your care planning), please list their first and last name to allow them to appear as a member of your “care team” on the dashboard.**
6. **Care partner designation—*Please indicate this person’s relationship to you. E.g.: “This person is my…”(spouse, child, parent, friend, other)***
7. **Needs Assessment—I would like a social worker to contact me for help with (check all that apply):**

0 = No needs at this time
1 = Transportation resources
2 = Support to help me cope with my illness and/or manage stress
3 = Financial concerns about my health care

1. **Needs Assessment—I would like a dietician to contact me to help with (check all that apply):**

0= No nutrition needs at this time
1= Significant gain or loss of weight without trying
2= Loss of appetite
3= Nausea / vomiting
4= Diarrhea
5= Constipation
6= Difficulty chewing or swallowing
7= Issues with my ostomy / feeding tube

1. **FACT-G7 (Version 4), GF7—I am content with the quality of my life right now...........**

0 = Not at all 1 = A little bit 2 = Somewhat 3 = Quite a bit 4 = Very much

1. **PROMIS Global01—In general, would you say your health is...........**

5 = Excellent 4 = Very good 3 = Good 2 = Fair 1 = Poor

1. **PROMIS Item Bank v1.0 - Dyspnea Severity (DYSSV014)—Over the past 7 days, how short of breath did you get with this activity….. standing for at least 5 minutes?**

0 = No shortness 1 = Mildly short 2 = Moderately short 3 = Severely short X I did not do this

of breath of breath of breath of breath in the past 7 days

1. **PRO-CTCAE 63a—In the last 7 days, were there times when you had to URINATE FREQUENTLY?**

0 = Never 1 = Rarely 2 = Occasionally 3 = Frequently 4 = Almost constantly

1. **PRO-CTCAE22b- Severity—In the last 7 days, what was the SEVERITY of your ARM OR LEG SWELLING at its WORST?**

0 = None 1 = Mild 2 = Moderate 3 = Severe 4 = Very Severe

1. **PROMIS Scale v1.0 - Gastrointestinal Nausea and Vomiting (GISX49)—How often did you have nausea—that is, a feeling like you could vomit?**

1 = Never 2 = Rarely 3 = Sometimes 4 = Often 5 = Always

1. **PROMIS Scale v1.0 - Gastrointestinal Nausea and Vomiting (GISX55)—How often did you have a poor appetite?**

1 = Never 2 = Rarely 3 = Sometimes 4 = Often 5 = Always

1. **PROMIS Item Pool v1.0 - Itch-Severity (PIQSeverity04)—How intense was your itch in general……….**

1 = Had no itch 2 = Mild 3 = Moderate 4 = Severe 5 = Very Severe

1. **FACT-GP5—I am bothered by side effects of treatment.**

0 = Not at all 1 = A little bit 2 = Somewhat 3 = Quite a bit 4 = Very much

- **PROMIS Item Bank v1.0 Fatigue (CAT administration)**
- **PROMIS Item Bank v1.0 - Emotional Distress - Depression (CAT administration)**
- **PROMIS Item Bank v2.0 - Physical Function (CAT Administration)**
