# Supplementary material for: Association Between a Co-Designed Dashboard and Use of Costly Health Services in Patients With Chronic Kidney Disease and Advanced Cancer: Propensity Score–Adjusted Difference-in-Differences Study
Source: J Med Internet Res. 2025 Nov 21;27:e70430. doi: 10.2196/70430 (PMC12680935; doi:10.2196/70430)
Supplement: Multimedia Appendix 2 [file jmir_v27i1e70430_app2.docx]

|  | Dashboard group | Comparison group |
| --- | --- | --- |
| Clinicians involved | 2 oncologists, 1 nephrologist, 1 nephrology PA, 2 primary care physicians at Northwestern Memorial Health Care | Same clinicians (for patients who chose not to enroll), or clinicians not participating in the dashboard study |
| Eligibility Criteria | **Advanced cancer** (stage 4 GI cancer, 3+ months IV chemotherapy; stage 3C/4 lung cancer, 3+ months chemo) **CKD** (stage 3 or eGFR < 60) | **Advanced cancer** (stage 4 GI cancer, 3+ months IV chemotherapy; stage 3C/4 lung cancer, 3+ months chemo) **CKD** (stage 3 or eGFR < 60) not exposed to the dashboard |
| Baseline Date | Date of initial dashboard questionnaire between June 8, 2020 and Nov 1, 2022 | **Advanced Cancer:** Date of first visit within study period **CKD:** First eGFR < 60 within study period |
| Observation Period | 6 months before and 6 months after each patient’s individualized baseline date | 6 months before and 6 months after baseline date defined above |
| Additional Criteria for CKD |  | Mean baseline eGFR calculated within +/- 30 days; exclude patients with eGFR > 60 (stage 2 CKD or lower) |
