# Supplementary material for: Association Between a Co-Designed Dashboard and Use of Costly Health Services in Patients With Chronic Kidney Disease and Advanced Cancer: Propensity Score–Adjusted Difference-in-Differences Study
Source: J Med Internet Res. 2025 Nov 21;27:e70430. doi: 10.2196/70430 (PMC12680935; doi:10.2196/70430)
Supplement: Multimedia Appendix 3 [file jmir_v27i1e70430_app3.docx]

|  | Dashboard group | | Comparison group | |  |
| --- | --- | --- | --- | --- | --- |
| Health Services Type | **Before** | **After** | **Before** | **After** | **Unweighted DiD β**  **(95% CI)** |
| Unplanned, all-cause hospital admissions | 57/284 (20.1%) | 63/284  (22.1%) | 157/917 (17.1%) | 244/917  (26.7%) | -0.074  (-0.151, 0.003) |
| EDAC within 30 days of hospital discharge | 116/284 (40.8%) | 129/284 (45.4%) | 143/917 (15.6%) | 219/917  (23.9%) | 0.027  (-0.014,0.069) |
| 7-day hospital readmissions | 4/284  (1.4%) | 14/284  (4.9%) | 4/917  (0.4%) | 5/917  (0.5%) | 0.034  (0.005, 0.064)* |
| Hospital admissions and ED visits for patients receiving outpatient chemotherapy | 30/284  (10.6%) | 54/284  (19.0%) | 43/917  (4.7%) | 161/917  (17.6%) | -0.044 (-0.108,0.020) |
| Oncology triage clinic use | 40/284  (14.1%) | 56/284  (19.7%) | 32/917  (3.4%) | 80/917  (8.7%) | 0.004  (-0.061, 0.069) |
| Completion of an advanced directive | 5/284  (1.7%) | 5/284  (1.7%) | 11/917  (1.2%) | 33/917  (3.6%) | -0.024  (-0.050, 0.002) |
| Hospice utilization^a^ |  | 11/27  (40.7%) |  | 32/65  (49.2%) | -0.085  (-0.312, 0.142) |

^a^ All coefficients are unadjusted treatment effects estimated using standard difference-in-differences (DiD) models. Linear β is the treatment-effect coefficient from a linear probability DiD. No baseline covariates were included in these models; estimates reflect unadjusted differences between treated and control groups across pre- and post-periods. For hospice utilization, which was only measured post-intervention, results represent a simple treated versus control contrast restricted to patients who died during the study period and who received care from a participating study physician. EDAC = excess (all-cause days); ED = Emergency Department.
*p<0.1; **p<0.05; ***p<0.01
