# Supplementary material for: Association Between a Co-Designed Dashboard and Use of Costly Health Services in Patients With Chronic Kidney Disease and Advanced Cancer: Propensity Score–Adjusted Difference-in-Differences Study
Source: J Med Internet Res. 2025 Nov 21;27:e70430. doi: 10.2196/70430 (PMC12680935; doi:10.2196/70430)
Supplement: Multimedia Appendix 4 [file jmir_v27i1e70430_app4.docx]

|  | Dashboard group | | Comparison group | |  |
| --- | --- | --- | --- | --- | --- |
| Health Services Type | **Before** | **After** | **Before** | **After** | **Unweighted DiD β**  **(95% CI)** |
| Unplanned, all-cause hospital admissions | 112/365  (30.7%) | 94/365  (25.8%) | 412/2137  (19.3%) | 385/2137  (18.0%) | -0.034  (-0.102, 0.034) |
| EDAC within 30 days of hospital discharge | 20/365  (5.4%) | 22/365  (6.0%) | 32/2137  (1.5%) | 23/2137  (1.1%) | 0.010  (-0.025,0.044) |
| 7-day hospital readmissions | 7/365  (1.9%) | 9/365  (2.4%) | 12/2137  (0.6%) | 10/2137  (0.5%) | 0.006  (-0.015, 0.028) |
| CKD-related ED or inpatient use | 128/365  (35.1%) | 112/365  (30.7%) | 483/2137  (22.6%) | 523/2137  (24.5%) | -0.063  (-0.135, 0.010) |
| Progression from CKD Stage 3 to 4, Stage 4 to Stage 5, and Stage 3 to Stage 5^a^ |  | 31/365  (8.4%) |  | 334/2137  (15.6%) | 0.014  (-0.016, 0.045) |

^a^ All coefficients are unadjusted treatment effects estimated using standard difference-in-differences (DiD) models. Linear β is the treatment-effect coefficient from a linear probability DiD. No baseline covariates were included in these models; estimates reflect unadjusted differences between treated and control groups across pre- and post-periods. Time and time*treated interaction term were excluded from the regression analyses. EDAC = excess (all-cause days); ED = Emergency Department.

*p<0.1; ** p<0.05; ***p<0.01
