# Supplementary material for: Association Between a Co-Designed Dashboard and Use of Costly Health Services in Patients With Chronic Kidney Disease and Advanced Cancer: Propensity Score–Adjusted Difference-in-Differences Study
Source: J Med Internet Res. 2025 Nov 21;27:e70430. doi: 10.2196/70430 (PMC12680935; doi:10.2196/70430)
Supplement: Multimedia Appendix 5 [file jmir_v27i1e70430_app5.docx]

|  |  | Dashboard group | | Comparison group | |  |
| --- | --- | --- | --- | --- | --- | --- |
| Cohort Type | **Health Services Type** | **Before** | **After** | **Before** | **After** | **Firth’s Adjusted Logit ATT ROR**  **(95% CI)** |
| Advanced Cancer | **7-day hospital readmissions** | 4/284  (1.4%) | 14/284  (4.9%) | 4/917  (0.4%) | 5/917  (0.5%) | 8.58  (2.28, 32.32)** |
| Advancer Cancer | **Completion of an advanced directive** | 5/284  (1.7%) | 5/284  (1.7%) | 11/917  (1.2%) | 33/917  (3.6%) | 0.25  (0.10, 0.67)** |
| CKD cohort | **Excess (all-cause) days in acute care (EDAC) within 30 days of hospital discharge** | 20/365  (5.4%) | 22/365  (6.0%) | 32/2137  (1.5%) | 23/2137  (1.1%) | 1.30  (0.72, 2.32) |
| CKD cohort | **7-day hospital readmissions** | 7/365  (1.9%) | 9/365  (2.4%) | 12/2137  (0.6%) | 10/2137  (0.5%) | 1.57  (0.65, 3.77) |

For very low sample prevalence (<10%), we additionally applied Firth’s penalized likelihood method to mitigate small sample bias.
