# Supplementary material for: Association Between a Co-Designed Dashboard and Use of Costly Health Services in Patients With Chronic Kidney Disease and Advanced Cancer: Propensity Score–Adjusted Difference-in-Differences Study
Source: J Med Internet Res. 2025 Nov 21;27:e70430. doi: 10.2196/70430 (PMC12680935; doi:10.2196/70430)
Supplement: Multimedia Appendix 6 [file jmir_v27i1e70430_app6.docx]

|  | Dashboard group | | Comparison group | |  |
| --- | --- | --- | --- | --- | --- |
| Health Services Type | **Before** | **After** | **Before** | **After** | **Linear ATT β (95% CI)** |
| Unplanned, all-cause hospital admissions | 57/284 (20.1%) | 63/284  (22.1%) | 157/917 (17.1%) | 244/917  (26.7%) | -0.093**  (-0.181, -0.014) |
| Excess (all-cause) days in acute care (EDAC) within 30 days of hospital discharge | 116/284 (40.8%) | 129/284 (45.4%) | 143/917 (15.6%) | 219/917  (23.9%) | 0.021  (-0.030, 0.073) |
| 7-day hospital readmissions | 4/284  (1.4%) | 14/284  (4.9%) | 4/917  (0.4%) | 5/917  (0.5%) | 0.044***  (0.011, 0.078) |
| Hospital admissions and ED visits for patients receiving outpatient chemotherapy | 30/284  (10.6%) | 54/284  (19.0%) | 43/917  (4.7%) | 161/917  (17.6%) | -0.087**  (-0.161, -0.012) |
| Use of the oncology triage clinic | 40/284  (14.1%) | 56/284  (19.7%) | 32/917  (3.4%) | 80/917  (8.7%) | -0.003  (-0.067, 0.062) |
| Completion of an advanced directive | 5/284  (1.7%) | 5/284  (1.7%) | 11/917  (1.2%) | 33/917  (3.6%) | -0.025**  (-0.049, -0.003) |
| Hospice utilization^a^ |  | 11/27  (40.7%) |  | 32/65  (49.2%) | 0.114  (-0.137, 0.366) |

^a^ All coefficients are the average treatment effect (ATE) obtained with inverse-propensity weighted difference-in-differences modes. Linear ATE β is the treatment-effect coefficient from the weighted linear probability DiD. The models adjust for all baseline covariates included in the propensity score specification to minimize residual confounding: race, ethnicity, age, sex, insurance category, Charlson Comorbidity Index, cancer type, baseline utilization encounter date, median household income (ZIP-code level), and baseline utilization counts (emergency, observation, inpatient, immediate/urgent care, and outpatient encounters). Time and time*treated interaction term were excluded from the regression analyses. For hospice utilization, the denominator is restricted to patients who died during the study period who received care from a participating study physician. EDAC = excess (all-cause days); ED = Emergency Department.

*p<0.1; ** p<0.05; ***p<0.01
