# Supplementary material for: Association Between a Co-Designed Dashboard and Use of Costly Health Services in Patients With Chronic Kidney Disease and Advanced Cancer: Propensity Score–Adjusted Difference-in-Differences Study
Source: J Med Internet Res. 2025 Nov 21;27:e70430. doi: 10.2196/70430 (PMC12680935; doi:10.2196/70430)
Supplement: Multimedia Appendix 7 [file jmir_v27i1e70430_app7.docx]

**=**

|  | Dashboard group | | Comparison group | |  |
| --- | --- | --- | --- | --- | --- |
| Health Services Type | **Before** | **After** | **Before** | **After** | **Linear ATE β (95% CI)** |
| Unplanned, all-cause hospital admissions | 112/365  (30.7%) | 94/365  (25.8%) | 412/2137  (19.3%) | 385/2137  (18.0%) | -0.002  (-0.079, 0.035) |
| Excess (all-cause) days in acute care (EDAC) within 30 days of hospital discharge | 20/365  (5.4%) | 22/365  (6.0%) | 32/2137  (1.5%) | 23/2137  (1.1%) | 0.003  (-0.024, 0.031) |
| 7-day hospital readmissions | 7/365  (1.9%) | 9/365  (2.4%) | 12/2137  (0.6%) | 10/2137  (0.5%) | 0.005  (-0.012, 0.022) |
| CKD-related ED or inpatient use | 128/365  (35.1%) | 112/365  (30.7%) | 483/2137  (22.6%) | 523/2137  (24.5%) | -0.043  (-0.107, 0.021) |
| Progression from CKD Stage 3 to 4, Stage 4 to Stage 5, and Stage 3 to Stage 5^a^ |  | 31/365  (8.4%) |  | 334/2137  (15.6%) | 0.007  (-0.013, 0.029) |

^a^ All coefficients are the average treatment effect (ATE) obtained with inverse propensity weighted difference-in-differences modes. Linear ATE β is the treatment-effect coefficient from the weighted linear probability DiD. The model adjusts for all baseline covariates included in the propensity score specification to minimize residual confounding: race, ethnicity, age, sex, insurance category, Charlson Comorbidity Index, CKD stage, baseline encounter date, median household income (ZIP-code level), and baseline utilization counts (emergency, observation, inpatient, immediate/urgent care, and outpatient encounters). Time and time*treated interaction term were excluded from the regression analyses. For hospice utilization, the denominator is restricted to patients who died during the study period who received care from a participating study physician. EDAC = excess (all-cause days); ED = Emergency Department.

*p<0.1; ** p<0.05; ***p<0.01
